# Supplementary material for: Relativistic magnetic reconnection driven by a laser interacting with a micro-scale plasma slab
Source: Nat Commun. 2018 Apr 23;9:1601. doi: 10.1038/s41467-018-04065-3 (PMC5913235; doi:10.1038/s41467-018-04065-3)
Supplement: Supplementary file 1 — Supplementary Information [file 41467_2018_4065_MOESM1_ESM.pdf]

Supplementary Information

for

"Relativistic Magnetic Reconnection Driven by a Laser Interacting with a Micro-scale Plasma Slab"

Yi et al.

Supplementary Note 1: Comparison simulation

Here we present a simulation with the setup depicted in Supplementary Fig. 1(a) as a comparison to the results shown in our main paper.

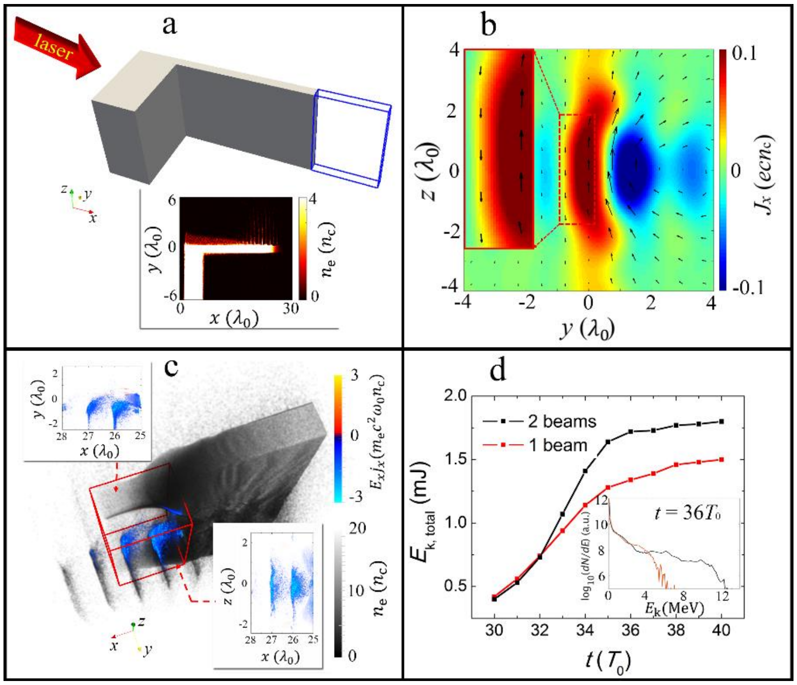

Supplementary Figure 1 | Schematic and main results from the comparison simulation with one laser-driven electron beam. (a) Sketch of the comparison simulation setup: a plate is used to block half of the laser pulse so that only one laser-driven electron beam reaches the corona (inside the blue box). The inset shows the electron density at cross-section $z = 0$ and simulation time $t = 30T_0$. (b) Longitudinal electric current density and transverse magnetic field (black arrows) at cross-section $x = 25\lambda_0$ and $t = 30T_0$, the zoom-in area shows the region with anti-parallel magnetic field configuration where MR may occur. (c) Field dissipation ($E_x j_x$) and electron density at $t = 33T_0$ in the corona for the one-beam simulation. (d) Time dependence of total electron kinetic energy for one-beam (red) and two-beams (black) cases, the inset shows the coronal electron energy spectra at $t = 36T_0$.

In this setup, an additional plate is placed on the $-y$ side of the plasma slab so that half of the laser beam is blocked. The plate thickness (in the longitudinal direction) is $5\lambda_0$ and has the same density ($20n_c$) as the slab. To ensure that the energy delivered to the corona [the volume within the blue box frame in Supplementary Fig. 1(a)] by the laser is roughly the same, we increase the laser amplitude by $\sqrt{2}$ ($a_0 = 7.07$). In this situation, only one electron beam reaches the coronal region as shown by the inset in Supplementary Fig. 1(a), and as a result the effects induced by MR are much weaker.

It should be noted that although almost all of the laser energy on the $-y$ side of the slab is blocked by the plate, the surface current on this side is, as shown by Supplementary Fig. 1(b), not exactly zero. Therefore MR may still occur in the area with an anti-parallel magnetic field configuration [zoom-in region in Supplementary Fig. 1(b)]. However, it is found that there are significant differences that support the arguments that we made in the paper, and are shown in Supplementary Fig. 1(c-d).

In Supplementary Fig. 1(c) we show the field dissipation ($E_x j_x$) in the corona in comparison with Fig. 4(a) in the paper. Obviously, the process is much stronger and significant in the 2-beams case. The reason is that the topology of the magnetic field lines is changed significantly when 2 electron beams from both sides of the slab reach the corona simultaneously. In contrast, when there is only one beam (even if it is approximately twice as intense), the collective motion of electrons, which extract energy from the collapse of magnetic fields, is almost negligible.

As a result, during the MR process, more magnetic energy is released and transferred to the plasma in the original 2-beams case, which is consistent with the analysis of electron energy in the corona, as shown in Supplementary Fig. 1(d). The total kinetic energy gain by the coronal electrons is 25% higher in the relativistic MR scenario proposed in the main paper. Moreover, we note a sharp increase of the $E_{\mathrm{k,total}}$ between $32T_0$ and $36T_0$ in the 2-beams case, which is in good agreement with the period during which the relativistic MR takes place. Moreover, the inset of Supplementary Fig. 1(d) shows the electron energy spectra at $t = 36T_0$ for two different cases. As one can see, the MR effect is mainly responsible for the generation of hot electrons which comprise the non-thermal tail in the spectrum.

Supplementary Note 2: Robustness of the mechanism

In order to demonstrate that the underlying physical process, the interaction of two laser-driven electron beams triggering relativistic MR, is a very robust mechanism and can be implemented with current experimental capabilities, we show an example based on a wire target instead of a slab in Supplementary Fig. 2. Such a target can be easily produced, and has already been used in previous experiments[1].

In the simulation the initial density of wire is $20n_{\mathrm{c}}$, the diameter is 8 μm (in *xy* plane), and the height is 28 μm (in *z* direction). A background plasma density with $0.01n_{\mathrm{c}}$ serves as the corona, which does not need to have an exponentially decreasing density profile as in the paper. Such underdense plasma may be provided by a gas jet, which has an exponential spatial distribution. However, for simplicity here we model it with a uniform density as the dimension of the simulation box is much smaller than the scale length of typical gas jet density distribution. The plasma is set to be pre-ionized and the physical ion/electron mass ratio is used. The size of the simulation box is $x \times y \times z = 20\lambda_0 \times 30\lambda_0 \times 30\lambda_0$, which is sampled by $400 \times 600 \times 600$ cells, with 5 macro particles for electron species and 3 for proton species.

Two laser pulses, with an angle of 10° in between, irradiate the wire from one side (front side), driving two electron beams which meet on the other side (back side). Each laser pulse has the same

parameters as the one used in the paper. Synchronization of two lasers can be challenging, but it has been demonstrated in previous experiments[2]. Moreover, if the laser spot size is larger than the diameter of the wire, one laser beam would be enough. Here we show the two laser case because it gives us the degree of freedom (temporal delay t_{d}) to perform some simple null tests.

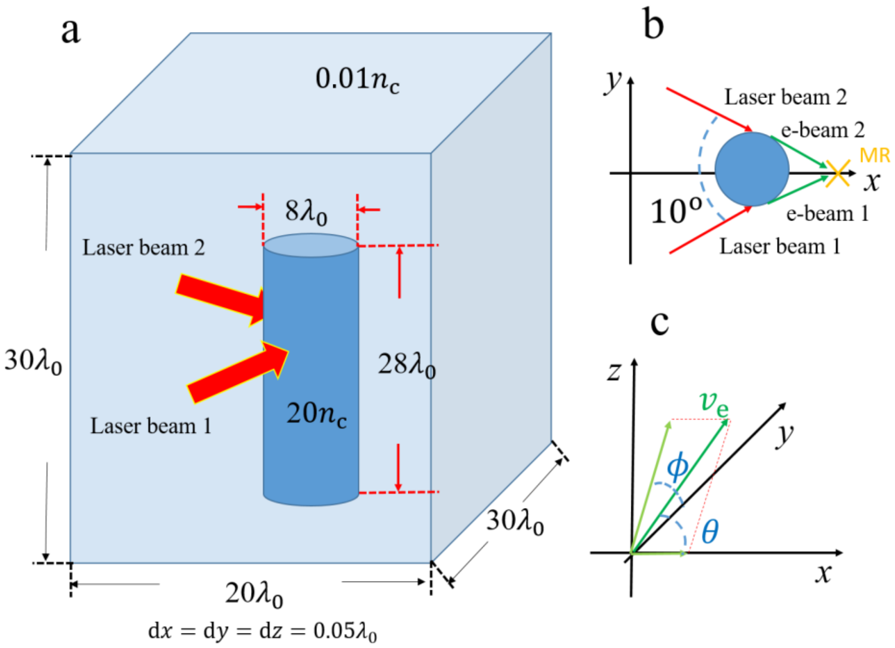

Supplementary Figure 2 | 3D sketch of the laser-wire MR setup. (a) Two laser pulses (red arrows) irradiate a wire on each side and drive two electron beams which triggers MR when they interact at the back side of the wire. The whole space (simulation box) is filled with underdense plasma (light blue). The simulation parameters are marked in the figure. The dark blue cylinder in the middle is a solid-density wire. (b) Cross section of the set-up in *xy* plane, where the laser-driven electrons and reconnection site are marked by green arrows and a yellow cross, respectively. (c) The coordinate system used in the simulation and the definition of the angles θ and ϕ, where v_{e} is the electron velocity, θ is the angle between v_{e} and $+x$ direction, and ϕ is the angle between the projection of v_{e} in the transverse plane (*yz*) with $+y$ direction.

By adjusting the temporal delay (t_{d}) of two laser pulses, three different simulations are performed for $t_{\mathrm{d}} = 0$, $t_{\mathrm{d}} = 0.5\tau$, and $t_{\mathrm{d}} = \tau$, respectively, where τ is the full duration of laser pulse. Note that the interaction of two laser-driven beams, and therefore MR, can only happen when $t_{\mathrm{d}} < \tau$. The backward propagating electrons with kinetic energy $E_{\mathrm{k}} > 4$ MeV are proposed as an experimental observable, as they can easily penetrate the wire target and be detected. The angular distribution and energy spectrum are shown in Supplementary Fig. 3.

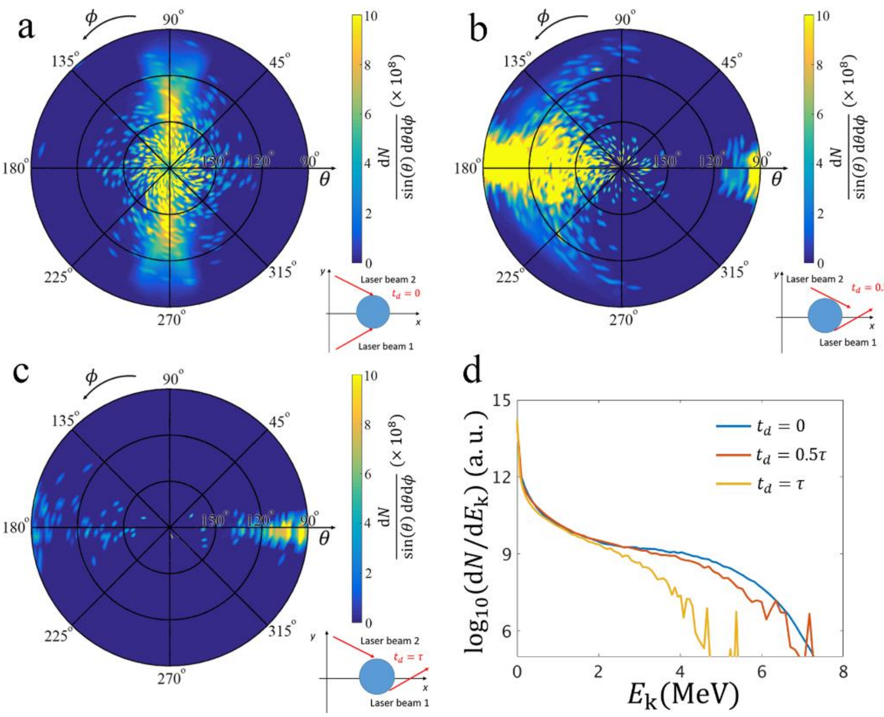

Supplementary Figure 3 | MR signatures from the laser-wire setup. Angular distribution of backward propagating ($P_x < 0$) electrons with kinetic energy $E_\mathrm{k} > 4$ MeV, for different temporal delays between the two laser pulses (a) $t_\mathrm{d} = 0$, (b) $t_\mathrm{d} = 0.5\tau$, and (c) $t_\mathrm{d} = \tau$. The insets in the bottom right of (a-c) show the corresponding sketch of the setup (cross-section in xy plane). (d) The energy spectrum of electrons emitted with $20^\mathrm{o} < \phi < 160^\mathrm{o}$ and $200^\mathrm{o} < \phi < 340^\mathrm{o}$ in three cases. The angles θ and ϕ are defined in Supplementary Fig. 2(c).

From Supplementary Fig. 3, one can see that there is a significant boost of the backward propagating electron energy and number when two laser beams temporally overlap, which allows the electron beams to interact with each other and trigger relativistic MR in the middle. The angular distribution pattern in the zero-temporal-delay case [Supplementary Fig. 3(a)] is extended in $\pm z$ direction ($\phi = 90^\mathrm{o}$ and 270°) due to the magnetic tension force. The pattern in Supplementary Fig. 3(b) is also leaning towards the $-y$ direction ($\phi = 180^\mathrm{o}$), because the interaction of the two beams is off axis. Supplementary Figure 3(c) shows a dramatic decrease of the backward propagating electrons when the temporal delay of two beams is greater than the duration, which indicates that the observed feature is a signature of relativistic MR. Finally, it should be noted that the reflection of the laser on the front side of the target will also produce backward propagating electrons, but they are emitted along $\pm y$ direction [shown by the bright spot around $\phi = 0^\mathrm{o}$ and 180° in Supplementary Fig. 3(b-c)], which can be easily separated in experiments. In Supplementary Fig. 3(d), we show the energy spectrum of the electrons which can be detected with $20^\mathrm{o} < \phi < 160^\mathrm{o}$, and $200^\mathrm{o} < \phi < 340^\mathrm{o}$ in the three cases. These results show very similar behavior of electron emission with slightly smaller cut-off energy (~ 8 MeV) than the setup presented in the paper. This demonstrates that the underlying mechanism is very robust.

Supplementary Note 3: Influence of laser pre-pulse and plasma density

To address the effects of a smooth vacuum-plasma interface (which might be induced by the laser pre-pulse) and the plasma density on the generation of magnetic fields, we conducted a 2D simulation with density 100 n_c and a smooth transverse target profile. The simulation box $x \times y = 30\lambda_0 \times 15\lambda_0$ is sampled by 3000×1500 cells, respectively, and the particle-per-cell is 50 for both protons and electron species. The transverse expansion profile is $n = n_0 \exp(-(y - y_0)^2/2\sigma_y^2)$, where $n_0 = 100n_c$ is the on-axis density between $\pm y_0$, and $y_0 = 0.25\lambda_0$. We show two cases with $\sigma_y = 0.25\lambda_0$ and $0.5\lambda_0$ in Supplementary Fig. 4(a-b) and Supplementary Fig. 4(c-d), respectively.

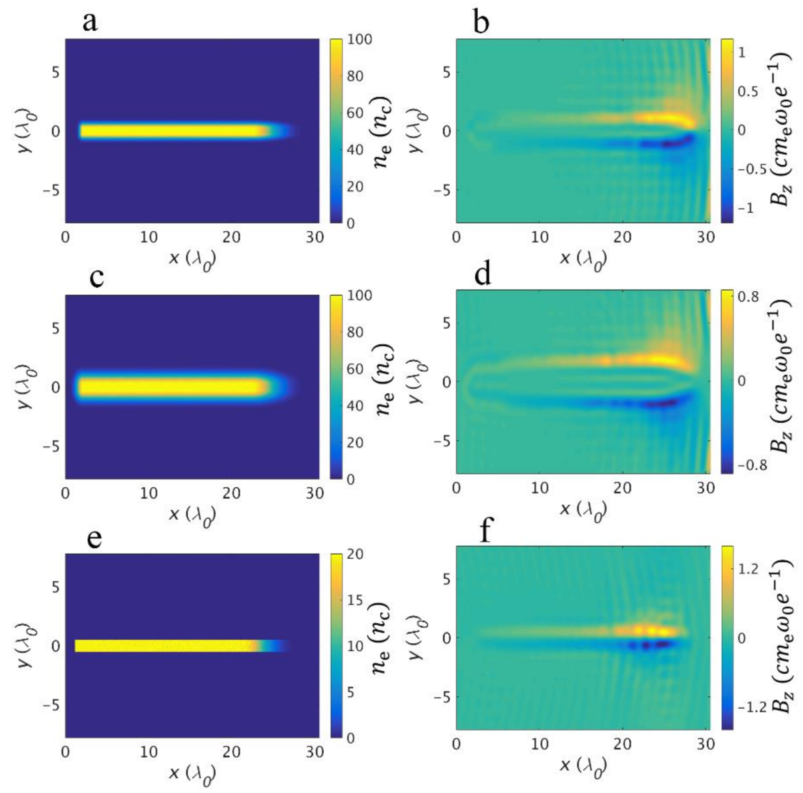

Supplementary Figure 4 | The initial density profile and static magnetic fields generated in 2D simulations. (a-b) $n_0 = 100n_c$, transverse scale length $\sigma_y = 0.25\lambda_0$; (c-d) $n_0 = 100n_c$, transverse scale length $\sigma_y = 0.5\lambda_0$; (e-f) shows the primary 3D simulation we used in the paper (2D cut at plane $z = 0$) with $n_0 = 20n_c$ and sharp transverse boundary.

Supplementary Fig. 4 shows that a smooth transverse profile and realistic $100n_c$ density does not significantly change the surface current and magnetic field generation process. The static magnetic fields in Supplementary Fig. 4(d) are about half the value in the 3D simulation with sharp boundary [Supplementary Fig. 4(f)], but this is mainly due to the increase of the effective slab thickness (defined as the transverse distance between two critical density layers), rather than due to the realistic $100n_c$ density or the smooth transverse profile. Therefore, as long as the transverse

expansion of the slab due to the laser pre-pulse is much smaller compared to the laser spot, it does not significantly change the results.

Supplementary References

1. Borghesi, M., *et al.*, Electric field detection in laser-plasma experiments via the proton imaging technique. *Phys. Plasmas* **9**, 2214-2220 (2002)

2. Aurand, B., *et al.*, Manipulation of the spatial distribution of laser-accelerated proton beams by varying the laser intensity distribution. *Phys. Plasmas* **23**, 023113 (2016)
